# Environmental passion and AI literacy shape the impact of green rewards on pro-environmental behaviors

**DOI:** 10.3389/fpsyg.2026.1699919

**Published:** 2026-02-25

**Authors:** Liya Xu, Yuyao Li, Tze-Haw Chan, Faiza Saleem

**Affiliations:** 1 Department of Development Planning, Shanxi Datong University, Datong, China; 2 Graduate School of Business, Universiti Sains Malaysia, Penang, Malaysia; 3 Faculty of Engineering, University of Malaya, Kuala Lumpur, Malaysia; 4 Department of Management Sciences, University of Wah, Wah Cantt, Pakistan

**Keywords:** AI literacy, environmental passion, green rewards, pro-environmental behaviors, Social Cognitive theory, Stimulus-Organism-Response theory

## Abstract

**Purpose:**

Considering the importance of pro-environmental behaviors in achieving Sustainable Development Goals, this paper examines and strengthens the understanding of how green rewards shape employees’ sustainable actions by integrating Stimulus-Organism-Response theory with Social Cognitive Theory. This paper explores the interplay between green rewards and pro-environmental behaviors. Specifically, this paper highlights the role of environmental passion as an internal mediating mechanism, and also analyzing the moderating influence of AI literacy as a critical capability in the digital era.

**Methods:**

This paper employed the Partial Least Squares Structural Equation Modeling combined with Artificial Neural Network analysis to capture both linear and nonlinear patterns in employee behaviors. The questionnaire respondents were from employees across various manufacturing and service sectors in China, with a total of 445 valid responses.

**Results:**

The results revealed that green rewards not only motivate pro-environmental behaviors but also foster positive environmental passion, which further enhance sustainable actions. Moreover, AI literacy significantly strengthens the positive influence of green rewards, amplifying their impacts on both environmental passion and pro-environmental behaviors. In addition, Artificial Neural Network analysis consistently identifies green rewards as the most influential predictor for both pro-environmental behaviors and environmental passion.

**Conclusion:**

These findings provided empirical support for the Stimulus-Organism-Response theory and the Social Cognitive theory. Results demonstrates that external incentives and internal psychological states jointly shape sustainable employee behaviors. To effectively promote pro-environmental behaviors, policymakers and managers should design targeted, sector-specific incentives and boost AI literacy.

## 1 Introduction

Achieving net-zero emission goals necessitates not only technological advancements and regulatory frameworks but also the pro-environmental behaviors of staff and customers (Zameer et al., 2021). Organizational initiatives take effect based on human action, including the adoption of low-carbon practices, waste reduction, and the innovation of greener processes are driven by individuals (Nawangsari et al., 2025). Sustainable development management and behavioral science advocate focusing on whether the efforts for sustainable development in companies and service environments are consistent with the motivations, capabilities, and behavioral cues of the Sustainable Development Goals (Mahajan et al., 2024). Pro-environmental behavior is defined as the action consciously aimed at minimizing environmental harm and is crucial for achieving sustainability goals (Andrzej et al., 2025). However, despite widespread awareness, current research in pro-environmental behaviors remains limited, necessitating innovative strategies to bridge the attitude-behavior gap (Sharma and Arora, 2025).

Within this paradigm, green incentives are important for fostering the change of pro-environmental behaviors. Green incentives include tangible rewards like financial compensation and points, and intangible recognition such as acknowledgment. Prior research indicates that rewards can foster sustainable practices by aligning individual incentives with environmental objectives (Wang and Guo, 2025). However, there are various opinions relating to the relationship between green rewards and pro-environmental behaviors. Maris et al. (2025) found that monetary incentives could not effectively change pro-environmental behaviors. Research of Xu et al. (2023) found that financial incentives in China’s recycling programs increased recycling rates but diminishing residents’ intrinsic motivation for environmental protection. Empirical research emphasized the reducing effect of financial incentives on social environmental norms of individuals (Ling et al., 2024). This effect is especially evident among groups that perceive themselves as environmentally sensitive or are profoundly embedded in their society. The contradictory viewpoints illustrate the necessity of further studying the relationship between green rewards and pro-environmental behaviors.

Despite increasing attention to the use of green rewards to pro-environmental behaviors, existing studies remain limited in two important respects. First, most research examines only the direct effect of green rewards on pro-environmental behaviors, without probing the underlying mechanisms (Akma et al., 2024; San Román-Niaves et al., 2025). Green rewards are an integral component of green human resource management (AlKetbi and Rice, 2024). Research of San Román-Niaves et al. (2025) identified that a shortcoming for current studies about the relationship between green human resource management and pro-environmental behaviors. They pointed out that studies have focused excessively on direct effects at the expense of in-depth exploration of causal pathways. Overlooking the potential mechanisms and possible contextual factors that may enhance or diminish their efficacy. Second, empirical evidence is heavily concentrated in household or community contexts, with relatively little work in organizational or sector-specific settings. Kusnawan et al. (2025) emphasized that study on pro-environmental behavior required expansion across all industries or types of organizations by looking through an analysis of 100 recent publications. Investigating manufacturing and service sectors is important because manufacturing field contends with resource-intensive processes, demanding compliance obligations, and the rising expectation for decarbonized supply chains (Lebepe and Mathaba, 2025). Service firms are characterized by their reduced material intensity and exert considerable influence over client interactions, potentially leading to broad behavioral consequences (Bonev, 2025). Digital technology and AI offer new opportunities to manufacturers and the service sector. Prior work highlights distinct environmental performance dynamics across these sectors (Cheng et al., 2024). Both sectors’ contrast provides an opportunity to understand how sector-specific conditions affect the impact of green rewards and related behavioral mechanisms.

To address these gaps, this study explores three potential mechanisms and contextual moderators, including green rewards, environmental passion, AI literacy. Environmental passion refers to affective and motivational emotion to protect the environment (Perano et al., 2025). Study of Saifulina et al. (2024) found that harmonious environmental passion is positively related to workplace pro-environmental behaviors. In addition, pro-environmental behaviors in private life could mediate their relationship. At the same time, passionate individuals may perceive rewards as validating their values rather than undermining autonomy (Mah et al., 2025). Environmental passion may be a major driving force in the relationship between reward and pro-environmental behaviors. However, few studies analyzed the environmental passion explicitly as a mediator in the link between rewards and pro-environmental behaviors, creating a gap that this study addresses.

Meanwhile, AI literacy refers to the knowledge and attitudes to apply AI tools (Muawanah et al., 2024). The development of AI also reshapes the context of behavior change (Divya et al., 2024). Higher AI literacy can make green rewards systems more effective by enabling employees to discover AI-assisted eco-efficiencies (Muawanah et al., 2024). However, disparities in literacy can diminish or skew the effectiveness of these reward mechanisms (Wang and Zhang, 2025). These complex relationships suggested that AI literacy may be a credible boundary condition for reward efficacy. In addition, the effects of AI literacy on the adoption of sustainable practices and consumption habits may be positive (Yu and Yi, 2024) or negative (Chittineni et al., 2025). How AI literacy acts as a moderator in influencing the rewards and pro-environmental behaviors remains uncertain.

This study fills significant gaps in the literature by developing a comprehensive paradigm that incorporates green rewards, environmental passion, AI literacy, and sectoral context. It is essential to comprehend not only that of green rewards but also the mechanisms and rationale behind it. Theoretically, it advances understanding of how motivational and technological factors jointly influence pro-environmental behaviors. This research elucidates the mediating effect of environmental passion and the moderating effect of AI literacy to shape employees’ responsiveness to green rewards. These contributions deepen the theoretical understanding of pro-environmental behaviors. Empirically, cross-sector comparison reveals how sector-specific pressures alter the effectiveness of green rewards. In addition, managers can design reward systems that match employee AI skills, use AI tools to amplify the impact of green rewards. Policy makers could tailor interventions to the specific challenges in manufacturing and service sectors.

In addition, there are several reasons for focusing on China. As the second largest emitter, China holds the ambitious obligation to protect the environment and ensure sustainability. China is committed to reaching peak emissions before 2030 and achieving carbon neutrality by 2060 (Cheng et al., 2024). China’s rapid development means that incentive-based behavioral strategies like offering green rewards can be a powerful tool for advancing its own environmental agenda and contributing to the worldwide climate solution. Significant improvements in environmental awareness and green behaviors are evident among the Chinese public. From 2003 to 2021, environmental values, knowledge, and concern notably increased, leading to more green purchasing and waste reduction (Zhang et al., 2024). These positive trends offer a strong foundation for studying how green rewards and environmental passion can further drive sustainable actions.

This paper employed PLS-SEM (Partial Least Squares Structural Equation Modeling) combined with Artificial Neural Network (ANN) to analyze the relationship between green rewards and pro-environmental behaviors, considering the mediating role of environmental passion and the moderating role of AI literacy. Based on these objectives, this paper addresses the following research questions:

*RQ1*: Does the provision of green rewards directly foster pro-environmental behavior?

*RQ2*: Do green rewards indirectly influence pro-environmental behavior by enhancing positive environmental passion?

*RQ3*: Does AI literacy moderate the relationship between green rewards and pro-environmental behavior?

*RQ4*: Could AI literacy promote the influencing mechanism of which green rewards increase environmental passion, then foster pro-environmental behavior?

The remainder of this paper is organized as follows. Section 2 outlines the research framework and formulates hypotheses by analyzing the recent two-year literature. Section 3 detailed the methodologies and data employed. Section 4 presents the results of PLS-SEM and ANN. Section 5 discusses these conclusions. Section 6 highlights the theoretical and practical implications of this study and offers future directions.

## 2 Literature reviews

### 2.1 Stimulus-Organism-Response theory

This paper employed the Stimulus-Organism-Response theory as the primary lens to conceptualize how green rewards of organizations translates into pro-environmental behaviors. Stimulus-Organism-Response theory posits that external stimuli (S) affect an individual’s internal state (O), which in turn shapes behavioral responses (R) (Maleknia and Enescu, 2025). In this study, green rewards function as the external stimulus that can alter employees’ internal motivational and emotional states, which in turn shape pro-environmental behaviors.

Recent applications of Stimulus-Organism-Response theory within the context of environmental and experience contexts further support its relevance for studying pro-environmental behaviors (Mansoor et al., 2025; Liu et al., 2025). The study of Mansoor et al. (2025) applies the Stimulus-Organism-Response framework to investigate how hotel green practices, individual perceptions, and social norms shape customer engagement in sustainable consumption, with pro-environmental behavior as the key outcome variable. It highlights the significant role of both external and internal stimuli in driving customers’ environmental actions. Liu et al. (2025) applied the Stimulus-Organism-Response model to digital consumption. Their research found that eco-information quality and platform transparency shaped consumers’ internal cognitive states, including perceived responsibility and environmental concern. Then consumers’ cognition ultimately driven green purchasing decisions. Similarly, Ilmalhaq et al. (2024) found that features such as green hotel design, visible eco-labels, and sustainability reminders act as external stimuli that boost tourists’ environmental emotions, which in turn increase their willingness to adopt low-carbon behaviors. These studies indicate that environmental stimuli consistently trigger affective and cognitive organismic states, which then translate into environmentally responsible actions. These findings demonstrate the structural explanatory power of the Stimulus-Organism-Response framework in green behavior research.

In addition, empirical researches chose organizational policies, incentives, and institutional cues act as external stimuli (Wang et al., 2024; Zhang et al., 2025). Zhou and Wu (2025) integrated Stimulus-Organism-Response theory and found that both positive and negative green imprints generate distinct emotional states that significantly influence green entrepreneurial behaviors. Their finding highlights the mechanism of Stimulus-Organism-Response theory. Research by Wang et al. (2024) identified information publicity, government transparency, and subjective norms as external stimuli indicators. These indicators activate cognitive evaluations and promote stronger pro-environmental behaviors. Zhang et al. (2025) emphasized the importance of rewards on tourists’ low-carbon behaviors. Aligning with these findings substantiates the notion that green rewards can function as an effective stimulus within the Stimulus-Organism-Response framework because green rewards shape individuals’ emotional and cognitive organismic states, which subsequently guide pro-environmental actions.

In addition, environmental passion is increasingly regarded as an organismic state that channels environmental stimuli into behavioral outcomes. A longitudinal study reveals that adolescents’ environmental passion enhances parental pro-environmental behaviors, with adolescent behavior and knowledge transmission serving as mediators of this relationship (Wang and Li, 2024). This emphasizes the importance of environmental passion as an internal state within the Stimulus-Organism-Response framework. Additional evidence further supports the mediating role of environmental passion in the Stimulus-Organism-Response framework. Kamil and Nordin (2025) found that a positive ethical climate during working acts as an external stimulus that increases employees’ environmental passion, which in turn mediates the effect on their low-carbon behaviors. These evidences reinforce that emotional and cognitive environmental states operate as key “organisms” in the Stimulus-Organism-Response process, such as environmental passion.

However, Stimulus-Organism-Response theory provides limited insight into how such organismic states are cognitively formed or why individuals respond differently to identical stimuli. To address this limitation, the present study incorporates Social Cognitive Theory to explicate the cognitive and self-regulatory mechanisms underlying the organismic component of the Stimulus-Organism-Response framework.

Sectoral differences are theoretically expected due to variations in task structure, technology use, and human-environment engagement across industries (Magliocca et al., 2024). Manufacturing sector features standardized processes and strong technological integration, which promotes consistent and technology-driven behaviors (Kovič et al., 2024). In contrast, service sectors demand more interpersonal interaction and judgment, which make behaviors more situation-dependent (Bian and Fan, 2024). Based on the Stimulus-Organism-Response theory, variations in sectoral characteristics may shape how organizational stimuli are interpreted, subsequently influence internal psychological states and observable behaviors. Standardized tasks and technological integration in manufacturing contexts can provide repeated learning opportunities that reinforces the relationship between stimulus and organism. By contrast, the variability and client-centric nature of service sectors may weaken or diversify these linkages by increasing reliance on contextual interpretation.

### 2.2 Social cognitive theory

Social Cognitive Theory is integrated in this study as a mechanism-oriented extension of the Stimulus-Organism-Response framework rather than as a parallel theoretical lens. Although the Stimulus-Organism-Response theory offers a useful macro-structure for understanding how stimuli influence internal states via a response, it is insufficient to explain the individual differences in cognitive processing and skill-based capacities that determine how stimuli are interpreted and translated into action. To address this, we integrate Social Cognitive theory, which emphasizes the role of cognitive competencies, knowledge, and self-efficacy in shaping behaviors. Social Cognitive theory explains how these internal states are cognitively constructed and enacted through learning, self-efficacy, and self-regulation (Yuan et al., 2024). Within the Stimulus-Organism-Response framework, external stimuli, such as environmental incentives or sustainability prompts, undergo internal processing before eliciting behavioral responses. From a social cognitive perspective, environmental passion reflects an affective–cognitive state shaped by learning experiences and self-regulatory engagement, rather than a purely emotional reaction. This perspective clarifies why environmental passion functions as a meaningful mediator between green rewards and pro-environmental behaviors.

Similarly, AI literacy aligns with the Social Cognitive Theory as a cognitive, capability-based moderator that helps explain heterogeneity in individuals’ responses to organizational incentives (Sarwar et al., 2025a). AI literacy can enhance these processes by boosting self-efficacy and improving the capacity to interpret digital environmental information (Almatrafi et al., 2024). AI literacy enables employees to more effectively translate external stimuli into action-relevant internal states. From a Social Cognitive Theory perspective, AI literacy interacts with incentive mechanisms such as green rewards to shape behavioral outcomes (Sarwar et al., 2024). Accordingly, AI literacy modifies how effectively green rewards are cognitively processed, thereby moderating the strength of the stimulus–organism linkage within the Stimulus-Organism-Response process.

From a social cognitive perspective, sectoral differences may influence the strength of both mediation and moderation mechanisms due to different learning opportunities, self-efficacy development, and self-regulatory engagement. Technology-intensive manufacturing contexts tend to foster task-specific training and tool-related self-efficacy (Subramanian and Suresh, 2024). This task-specific environment could strengthen cognitively grounded organismic states. On the contrary, service contexts exhibit greater heterogeneity in capability development, resulting in more variable mediation and moderation effects.

Combining Stimulus-Organism-Response theory and Social Cognitive theory yields a richer theoretical account. Stimulus-Organism-Response theory provides the overarching causal structure of the proposed model, while Social Cognitive Theory deepens the explanation of the organismic mechanisms through which mediation and moderation occur. This integrated framing is particularly appropriate for current research. It captures how environmental passion mediates and AI literacy moderates the relationship between green rewards and green behaviors.

### 2.3 Green rewards and pro-environmental behaviors

Green incentives include tangible rewards like financial compensation and points, and intangible recognition such as acknowledgment (Sarwar et al., 2025b). There are numerous studies supporting that green rewards can trigger measurable increases in pro-environmental behaviors. Study of Kroker and Lange (2024) demonstrates that financial incentives enhance significant pro-environmental behaviors in the laboratory, such as energy conservation or charity contributions to environmental organizations. In addition, they analyzed the impact of financial incentives compared to public welfare incentives on pro-environmental behaviors in a specific laboratory environment. Their findings also indicated that financial incentives were more effective than public welfare incentives in promoting pro-environmental behaviors, and the existence of a time-sensitive effect on behavioral modifications prompted by incentives demonstrated. Wang and Guo (2025) found that the attainment of gamified green points during gaming and hotel accommodations increases customers’ connection to nature and environmental awareness. Furthermore, Yang et al. (2024) found that threshold-based incentive schemes more efficiently encourage pro-environmental behaviors than universal rewards. Individuals receive benefits solely upon achieving designated environmental performance levels, hence fostering more robust environmental action than immediate direct payments. They pointed out the key mechanism of this incentive is to augment individuals’ environmental self-efficacy. Similarly, researches of Yin et al. (2025) and Sasaki et al. (2025) also supported the positive effect of green rewards on pro-environmental behaviors. These evidences confirm the positive impact of specific green rewards mechanisms on pro-environmental behaviors within a single environment.

However, scholars have proposed opposite results regarding the relationship between green rewards and pro-environmental behaviors. Andrzej et al. (2025) compared the effects of minor financial incentives on pro-environmental behaviors in the United States and the United Kingdom. The research indicated that minor financial incentives significantly influenced long-term environmental behavior in the United Kingdom, whereas they had no notable effect in the United States. Maris et al. (2025) also found that monetary incentives could not effectively change pro-environmental behaviors. Based on the above contradictory conclusions, this study puts forward the following hypotheses.

*H1*: Green rewards boost the pro-environmental behaviors.

### 2.4 The mediating role of environmental passion

Environmental passion refers to a vibrant, positive emotional state characterized by passion and a strong orientation toward safeguarding the environment (Perano et al., 2025). Relevant studies found that the positive or negative relationships between green rewards and environmental passion. Research by Yang et al. (2024) indicates that incentives for advancing achievement might amplify environmental passion by offering feedback, improving self-efficacy, or illustrating the green societal value. However, Nyanghura et al. (2024) revealed that monetary or other external rewards can undermine eco-friendly motivation when they are perceived as inconsistent with an individual’s core values. Such rewards may reduce environmental passion once the incentives are removed. Considering the complexity of environmental emission and green rewards, this paper proposes the following hypotheses:

*H2a*: Emphasizing green rewards can boost environmental passion.

Environmental passion is widely associated with stronger pro-environmental behaviors. This profound environmental passion acts as a significant catalyst, encouraging individuals to adopt pro-environmental behaviors and motivating them to further contribute constructively to ecological preservation (Wang and Li, 2024). Wang and Li (2024) examined the environmental passion of adolescents and the pro-environmental behaviors of their parents. Their research revealed a positive correlation between the two variables and identified that the family conversation pattern exerts a reinforcing influence on their association. Nguyen and Nguyen (2025) found a similar effect of environmental passion on pro-environmental behaviors of students in a university. However, the interaction of adolescent environmental passion with family conformity patterns could negatively influence parents’ pro-environmental behaviors (Wang and Li, 2024). Similarly, Saifulina et al. (2024) found the positive and significant relationship between harmonious environmental passion and voluntary pro-environmental behaviors at home and in the workplace. Moreover, research of Niu et al. (2025) found that both positive and negative expected emotions influence pro-environmental behaviors, with positive emotions having a direct effect.

Although many studies support that positive environmental passion foster pro-environmental behaviors, there is also growing evidence suggesting that certain conditions may undermine this effect. A recent study showed that increasing farming income can promote farmland conservation practices in the short term (Li et al., 2025a). However, they pointed out that crowding-out effect of intrinsic motivation would raise doubts about this long-term compliance. In addition, a quasi-experimental investigation of household recycling in China revealed that opposition to eco-friendly societal norms is more obvious among individuals with a strong environmental self-identity (Ling et al., 2024). Given these complexities, this study recognizes that the mechanism of which environmental passion promotes pro-environmental behaviors may not always hold across contexts. Therefore, this study designs the part of the second hypothesis.

*H2b*: Positive environmental passion enhances the pro-environmental behavior.

Considering the dual relationships of environmental passion with green rewards and pro-environmental behaviors, this paper employed Stimulus-Organism-Response theoretical framework for studying series of relationships. A recent study indicated that financial incentives can temporarily enhance specific environmentally friendly activities (Alt et al., 2024). Nevertheless, the retraction of these incentives may induce adverse effects within the target demographic, inhibiting additional voluntary environmental initiatives. Similar issues were posed in research of Nyanghura et al. (2024). Nyanghura et al. (2024) showed that green rewards might diminish environmental passion when they are regarded as coercive or incongruent with an individual’s principles. This weakening in turn lowers the probability of initiating or maintaining environmentally protective activities. Building on these findings, the present study proposes that environmental passion mediates the effect of green rewards on pro-environmental behaviors, capturing both the positive motivational impact of appropriate green rewards and the potential attenuation when green rewards undermine environmental passion. This paper posed the following hypothesis H2, which integrates H2a and H2b to test whether green rewards influence pro-environmental behaviors through the mediating role of environmental passion.

*H2*: Green rewards could increase positive environmental passion, and then positive environmental passion enhance the pro-environmental behaviors.

### 2.5 The moderating role of AI literacy

AI literacy refers to the employees’ domain-specific capability to understand, evaluate, and effectively apply AI technologies in the workplace (Zehui et al., 2025). Recent research provides supporting evidence that AI literacy is beneficial for the green rewards. Research by Yu and Yi (2024) indicated a strong correlation between higher digital literacy and increased adoption of sustainable practices and consumption habits. This suggested that digitally proficient users were better equipped to leverage the value from reward systems integrated into apps or organizational tools. AI literacy provided individuals with green applications and platforms that offered incentives like rewards, points, or badges (Muawanah et al., 2024). However, disparities in literacy can diminish or skew the effectiveness of these reward mechanisms. A study of Wang and Zhang (2025) found that uneven capacity to utilize green technologies could reduce the responses to incentives among less digitally literate populations. In addition, Kim and Kim (2025) found that the AI integration of organizations has been linked to reduced pro-environmental participation when associated with information overload risks. Their research implied that poorly conceived technological frameworks aligning with reward systems might inadvertently divert attention from environmental objectives. The importance and uncertainty of the correlation between AI literacy and green rewards are highlighted by this research.

Multiple studies link higher AI literacy with pro-environmental behaviors across various fields, including consumer behaviors and farm production. Pataranutaporn et al. (2025) introduced a conversational AI system that utilizes animated marine characters to encourage users toward sustainable behaviors. Their research revealed that participants interacting with these AI-driven characters exhibited significantly stronger behavioral intentions and preferences for sustainable choices compared to those exposed to static information. Research of Liao (2025) showed that perceived usefulness, ease of use, and self-efficacy concerning AI products directly predict the behavioral intention to use environmental AI products, then promoting pro-environmental behaviors. However, the effects of AI literacy on pro-environmental actions are not uniformly positive. Study of Chittineni et al. (2025) demonstrated that the frequency of GenAI usage is negatively associated with environmental responsibility and energy-efficient practices. AI adoption would increase the energy consumption (Desroches et al., 2025). Literacy gaps can produce unequal environmental outcomes.

According to Social Cognitive theory, an individual’s pro-environmental behavior is impacted by external incentives as well as by their cognitive assessment of personal capabilities and the situational clues offered by technology (Yuan et al., 2024). AI literacy enhances these processes by boosting self-efficacy and improving the capacity to interpret digital environmental information (Almatrafi et al., 2024). Recent studies indicate that AI can enhance or control pro-environmental behaviors (Chittineni et al., 2025; Liao, 2025). In addition, AI literacy has positive or negative relationships with incentives (Muawanah et al., 2024; Kim and Kim, 2025). From a Social Cognitive theory perspective, AI literacy may operate as a moderator that interacts with incentive factors to influence behavior. Accordingly, this study proposes that AI moderates the relationship between green rewards and pro-environmental behaviors. The hypothesis is designed as follows.

*H3*: AI literacy positively moderates the relationship between green rewards and pro-environmental behaviors.

Recent research in sustainability education indicates that AI-supported learning can deepen environmental understanding and engagement (Arif et al., 2025). Study of Leal et al. (2025) revealed that education aligning with AI tools is positively associated with sustainability literacy and environmental awareness. However, Ruiu et al. (2025) discovered that literacy shortfalls and ethical or privacy concerns around AI can dampen trust and reduce willingness to emotionally invest, especially when users feel disempowered by opaque systems. Given the mixed findings above, treating AI literacy as a moderator is warranted to explain when rewards enhance passion into behaviors is still unexplained. In consideration of the above analysis, this article constructs the following hypothesis.

*H4*: AI literacy positively moderates the relationship between green rewards, environmental passion, and pro-environmental behaviors.

### 2.6 Conceptual framework

**Figure 1:**
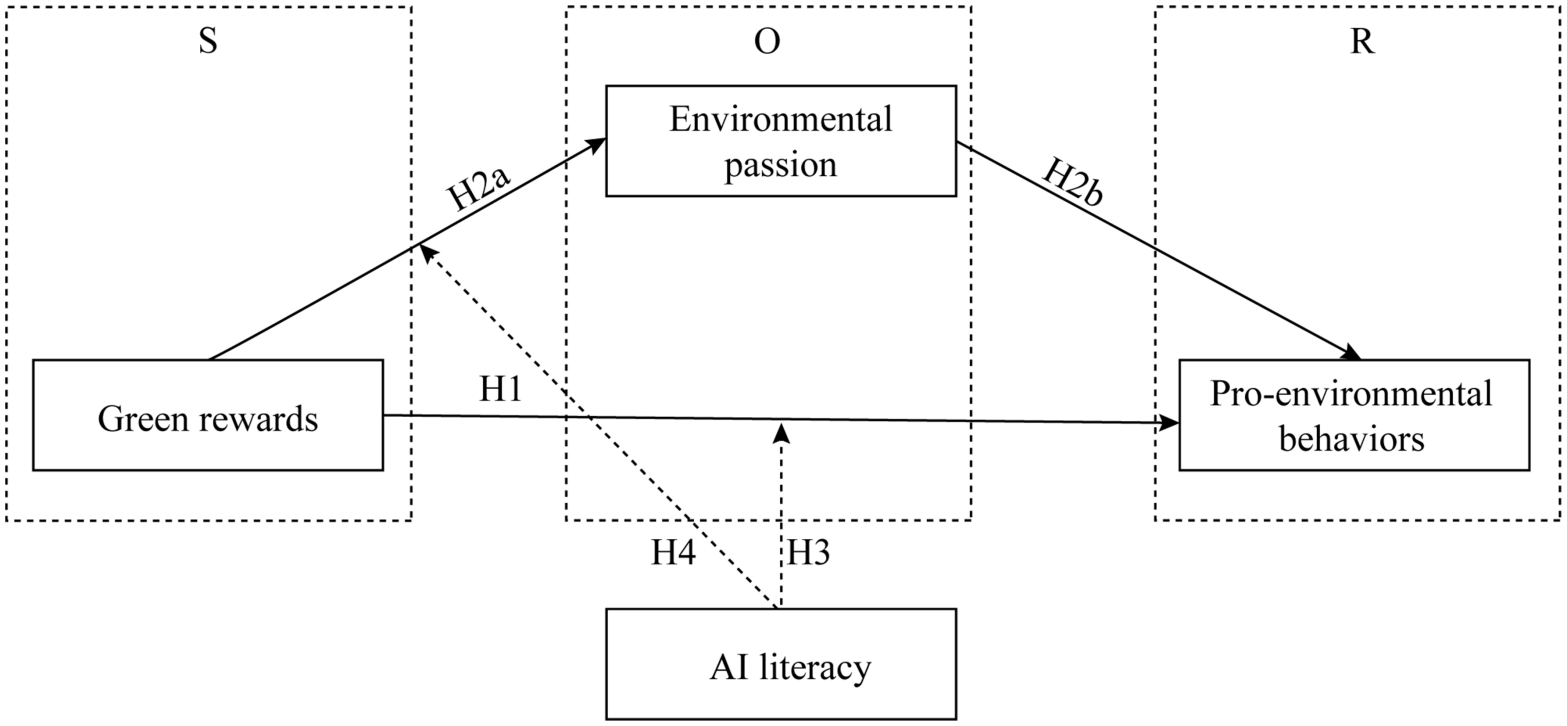
Conceptual framework. A conceptual framework diagram illustrating the stimulus–organism–response (SOR) model. The stimulus (S) is “Green rewards,” which has a direct path to “Pro-environmental behaviors” (H1) and an indirect path through “Environmental passion” (H2a and H2b). “Environmental passion” represent the organism (O) and lead to “Pro-environmental behaviors,” the response (R). “AI literacy” moderates two relationships: between “Green rewards” and “Pro-environmental behaviors” (H3) and between “Green rewards” and “Environmental passion” (H4).

This study presents a conceptual framework aimed at examining the influencing mechanism of pro-environmental behaviors (Figure 1). Based on the Stimulus-Organism-Response theory, the framework argues that green reward functions as external incentives, environmental passion acts as an internal state, and then pro-environmental behavior is a behavioral response. H1 and H2 are designed to examine the direct effect of green rewards and the mediating effect of environmental passion in influencing the relationship between green rewards and pro-environmental behavior. H2 integrates H2a and H2b to test whether green rewards influence pro-environmental behaviors through the mediating role of environmental passion.

However, the strength of these relationships may differ among individuals. Based on the social cognitive theory, this paper suggests that AI literacy influences the impact of both green rewards and environmental passion on pro-environmental behavior. To account for this variability, the framework incorporates AI literacy as a moderating variable and designs H3 and H4 to examine the moderating effects of AI literacy in relationships between green rewards, environmental passion, and pro-environmental behaviors.

## 3 Materials and methods

### 3.1 Data sources

All variables are measured and accessed by question items. The dependent variable is pro-environmental behavior. This paper selected the questions from Zhang and Sun (2024), which mainly measure whether the respondents take the initiative to adopt pro-environmental behaviors. This study chose green rewards as the independent variable, measuring through questions from Saputra et al. (2024) and Das and Dash (2024). To assess the environmental passion, this paper chose the question items constructed by Gregori et al. (2024). Furthermore, this study utilized 11 items from Wang B. et al. (2023) to evaluate the AI literacy, from the perspectives of AI awareness, usage, evaluation, and ethics. First, AI awareness reflects employees’ basic recognition and understanding of AI technologies in the workplace (Sari et al., 2025). This dimension captures whether employees can distinguish AI-enabled devices from non-smart technologies, and understand how AI technologies can support their work tasks. AI awareness is measured using three items of the scale (AL1–AL3), as listed in the questionnaire provided in the Supplementary materials. Second, AI usage captures employees’ capability to effectively apply AI applications or products in daily work activities (Muehlemann, 2025). This dimension is measured through five items of the scale, including AL4–AL6 and AL8–AL9. Third, AI evaluation denotes employees’ capacity to critically assess AI technologies after practical use (Benlian and Pinski, 2025). This dimension reflects whether employees can evaluate the capabilities and limitations of AI applications, as measured by item AL7. Forth, ethical awareness emphasizes responsible and cautious AI use (Ho and Lee, 2025). This dimension is measured by item AL10 and AL11.

To mitigate potential common method bias, several procedural remedies were implemented. First, all measurement items were adapted from well-established questions constructed by Zhang and Sun (2024), Saputra et al. (2024), Das and Dash (2024), Gregori et al. (2024), and Wang B. et al. (2023). These scales have been extensively applied in the relevant literature and demonstrated satisfactory reliability and validity across different contexts. The adoption of maturity scales could reduce measurement error and item ambiguity. Secondly, respondents received assurances of anonymity and confidentiality, which helped to reduce evaluation apprehension and social desirability bias. Furthermore, questionnaires are carefully designed using neutral wording.

### 3.2 Data collection

This study aims to examine the correlations among green rewards, environmental passion, AI literacy, and pro-environmental behaviors in the manufacturing and service sectors. The intended respondents comprise employees who possess familiarity with and an intimate understanding of contemporary advancements and AI tools in manufacturing and service sectors. Because the research focuses on employee-level behavioral mechanisms rather than sector-specific differences, the survey did not restrict participation to particular subsectors within manufacturing or services.

The questionnaire was distributed online to employees in Shanghai and Beijing through digital channels and peer sharing. Data of this study was collected using Wen Juanxing software. This software is a widely used and ISO-certified online survey platform in China. The platform complies with national data protection regulations and provides encrypted data transmission, anonymous response options, and secure cloud storage (Li et al., 2025b). All respondents participated voluntarily and anonymously, and the survey procedure adhered to the ethical guidelines of the authors’ institutions. No personal identifying information was collected. Existing studies have used this software to conduct analysis about China (Su et al., 2024; Li et al., 2025b; Zhao et al., 2025). Before data analysis, a data quality screening procedure was conducted. First, 15 responses with incomplete or missing information were removed. Second, 4 cases with abnormal completion times were excluded to ensure response reliability, following common practice in online survey quality control (Zhao et al., 2025). After these procedures, a total of 455 valid responses remained for analysis. The full questionnaire used in this study is publicly available, as indicated in the Supplementary materials.

Although part of measurement scales was originally developed in Western contexts, their applicability to the Chinese organizational setting is well supported. First, the constructs examined in this study capture general cognitive and behavioral processes rather than culture-specific practices. Second, the original English scales were translated into Chinese using a translation–back-translation procedure to ensure semantic and conceptual equivalence. Third, the translated questionnaire was carefully reviewed to ensure clarity and contextual appropriateness before formal data collection. Finally, the reliability and validity of the measurement scales were assessed and are reported in the Results section, providing empirical support for their suitability in the Chinese sample.

Due to the open distribution and voluntary participation nature of the study, convenience sampling combined with snowball sampling was employed. Convenience sampling is widely recognized as an appropriate approach for exploratory behavioral research (Ilker et al., 2015). Addition of snowball sampling is necessary to expand respondent reach within professional networks, aligning with recent methodological recommendations for survey studies in business and management (Ting et al., 2025). While these non-probability sampling techniques limit full generalizability, the sample size and diversity across demographic categories enhance the study’s external validity to a reasonable extent for exploratory research. This is also supported by Shaukat et al. (2024). Recent discussions in organizational research also highlight that non-probability sampling is increasingly appropriate and widely used in digital survey environments, where access to fully randomized employee samples is often infeasible (Zickar and Keith, 2023).

Furthermore, a G*Power analysis was conducted to justify the adequacy of the sample size (Moshagen and Bader, 2024). Using a medium effect size (*f*^2^ = 0.15), an alpha level of 0.05, and statistical power of 0.80 for a model with four predictors, the minimum required sample size was 85 (Soliman et al., 2024). The collected sample of 445 respondents therefore exceeds the recommended threshold, supporting sufficient statistical power for the analyses.

To provide a clearer understanding of the sample, a sample profile before PLS-SEM analysis would be added, including respondents’ gender, ownership structure, working ages, educational background, number of employees, and type of industry. This profile helps illustrate the diversity of respondents and the coverage (Gao et al., 2025).

All survey questions are measured by a 7-point Likert scale. While both 5-point and 7-point formats are commonly used in pro-environmental behavior research, a 7-point scale allows respondents to express attitudes with finer gradations and has been shown to improve scale sensitivity and reliability in complex behavioral constructs (Gao et al., 2025; Finstad, 2010). The 7-point scale provides adequate variability for detecting subtle differences in respondents’ perceptions and reactions (Tian et al., 2024). This paper rates items using the following scale: 1 = strongly disagree, 2 = disagree, 3 = somewhat disagree, 4 = neutral, 5 = somewhat agree, 6 = agree, and 7 = strongly agree.

### 3.3 PLS-SEM modeling

The PLS-SEM is the most appropriate analytical technique for investigating the impact of green rewards on pro-environmental behaviors via environmental passion and under the moderating effect of AI literacy, particularly in contrast to regression models or Covariance-Based SEM (CB-SEM). This choice aligns with recent methodological guidelines emphasizing prediction-oriented, theory-development research in behavioral and organizational studies (Guenther et al., 2023).

Compared to the regression, PLS-SEM effectively manages latent constructs and incorporates measurement error (Hair et al., 2021). Regression requires composite scores and is incapable of modeling relationships among unobserved variables. Recent studies further highlight that regression becomes increasingly inadequate when mediation and moderation are analyzed simultaneously, as it cannot estimate measurement and structural relationships within one unified model (Olaniyi, 2025). However, PLS-SEM concurrently estimates both measurement and structural models, effectively capturing intricate indirect effects (Soliman et al., 2024).

Compared to the CB-SEM, PLS-SEM exhibits enhanced efficacy in explaining behavioral research. It requires fewer distributional assumptions and produces reliable findings with moderate sample sizes (Vuković, 2024). PLS-SEM offers stronger predictive assessment through variance-based criteria. Current evidence confirms that PLS-SEM is superior when data deviate from normality or when constructs involve formative or interaction terms (Magno et al., 2024). PLS-SEM provides a robust predictive capability through analyzing variance in endogenous factors (Misa et al., 2024). Moreover, PLS-SEM is suitable for estimating models that incorporate mediation and moderation (Sarstedt and Moisescu, 2024). Its algorithm facilitates product-indicator and two-stage approaches for moderation, providing more enhanced flexibility than CB-SEM. PLS-SEM is suitable for this study, using environmental passion as a mediator and AI literacy as a moderator.

In addition, this paper employed Pearson’s correlation coefficients to examine preliminary relationships among green rewards, environmental passion, AI literacy, and pro-environmental behaviors. Then, all main hypotheses were further tested using PLS-SEM analysis to test the hypothesized structural model. The test sequence is consistent with recent best practices by Hair et al. (2022b) and Pereira et al. (2024). Questionnaire data are first described via correlational analysis, and then main hypotheses and structural relationships are tested using PLS-SEM.

### 3.4 ANN modeling

Integrating an ANN with PLS-SEM significantly enhances analytical depth by effectively capturing complex nonlinear relationships that PLS-SEM alone cannot fully address. Although PLS-SEM effectively estimates linear connections and clarifies causal pathways, including mediation and moderation, human and organizational behaviors often display nonlinear effects that exceed its detection capabilities. More importantly, PLS-SEM primarily focuses on theory confirmation through linear path significance, whereas ANN enables a deeper exploration of how strongly and in what manner different predictors contribute to the outcome. Combining ANN with PLS-SEM allows for the identification of these intricate nonlinear patterns, thereby improving predictive accuracy and quantifying relative importance (Sun et al., 2024). The relative importance derived from ANN could refine construct prioritization within the proposed framework, offering insights into which factors play a more dominant role beyond what can be inferred from standardized path coefficients alone.

Recent research employed a two-stage SEM-ANN procedure to analyze potential factors that could influence students’ e-learning continuance intention and operational performances (Xu et al., 2024; Elsherbeny et al., 2025). These studies confirmed the validity and improved the predictive performance of the ANN model, which was developed following the application of PLS-SEM. Specifically, PLS-SEM is utilized in the initial phase to establish the theoretical structural relationships, while the ANN is subsequently applied for prediction and sensitivity analysis. This hybrid approach provides a foundation for more comprehensive and robust research outcomes. This increasingly recognizes that the combination of PLS-SEM and ANN analysis in behavioral research offers a powerful combination of explanatory rigor and practical predictive precision (Leong et al., 2024). Accordingly, ANN in this study is not employed merely as a robustness or validation tool, but as a complementary analytical approach that provides additional theoretical insights into the relative influence and underlying complexity of the proposed relationships.

**Figure 2:**
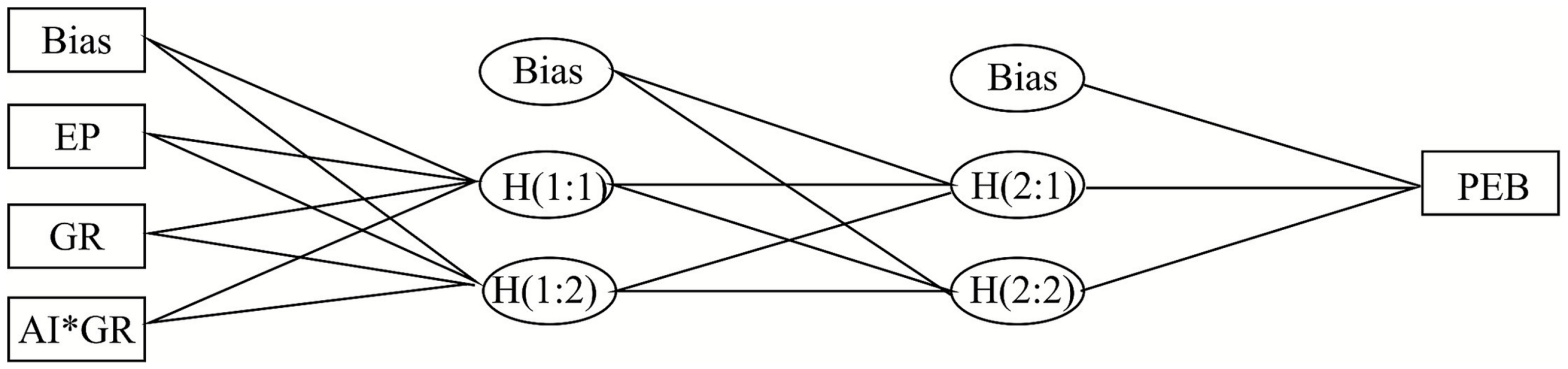
ANN architecture for pro-environmental behavior predicting. Bias = Bias inputs; EP = Environmental Passion; GR = Green Reward; AI* GR = Interaction term between AI literacy and GR; H1:1, H1:2 = First-layer hidden nodes; H2:1, H2:2 = Second-layer hidden nodes; PEB = Output node representing pro-environmental behavior. An ANN model diagram showing the relationships leading to pro-environmental behavior (PEB). Inputs include Bias, Environmental Passion (EP), Green Reward (GR), and the interaction term AI*GR. These variables connect to two first-layer hidden nodes (H1:1 and H1:2), which then link to second-layer hidden nodes (H2:1 and H2:2), along with additional bias terms. The second-layer nodes lead to the output node representing PEB.

**Figure 3:**
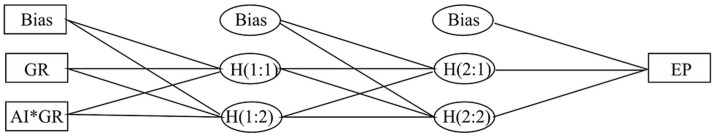
ANN architecture for environmental passion predicting. GR = Green Reward; AI*GR = Interaction term; Bias= Bias inputs; H1:1, H1:2 = First-layer hidden nodes; H2:1, H2:2= Second-layer hidden nodes; EP= Environmental Passion (output). Description: An ANN model diagram showing the relationships leading to Environmental Passion (EP). Inputs include Bias, Green Reward (GR), and the interaction term AI*GR. These variables connect to two first-layer hidden nodes (H1:1 and H1:2), which then link to second-layer hidden nodes (H2:1 and H2:2), along with additional bias terms. The second-layer nodes lead to the output node representing EP.

The following Figures 2, 3 show the designed framework for analyzing pro-environmental behaviors, environmental passion through ANN. AI*GR in figures refers to the interactive term of AI literacy, aligning with green rewards.

## 4 Results

### 4.1 Demographic information and correlation

Table 1 lists all demographic information of respondents, including gender, ownership structure, working ages, educational background, number of employees, and type of industry. The classification of gender and educational background sources from the research of Wang B. et al. (2023). The classification of ownership structure and industry is based on the research of Tang et al. (2018). This study adopts the classification of working age and employees’ numbers from Gao et al. (2025).

**Table 1:** Demographic information.

Demographic	Description	Frequency	%
Gender	Female	223	50.11%
	Male	222	49.89%
Ownership structure	State owned	135	30.34%
	Privately owned	216	48.54%
	Foreign owned	33	7.42%
	Joint venture	61	13.71%
Working ages	Less than 5 years	154	34.61%
	5–10 years	133	29.89%
	11–20 years	132	29.66%
	More than 20 years	26	5.84%
Educational background	Middle school	32	7.19%
	High school	62	13.93%
	Bachelor	210	47.19%
	Master	91	20.45%
	Ph. D	50	11.24%
Number of employees	1–20	56	12.58%
	21–50	164	36.85%
	51–80	178	40.00%
	Above 80	47	10.56%
Type of industry	Manufacturing	235	52.81%
	Service	210	47.19%

Sources: summary by the author.

The demographic profile shows a balanced gender distribution, with females (50.11%) and males (49.89%). Most firms are privately owned (48.54%), followed by state-owned enterprises (30.34%). Foreign-owned and joint venture firms are 7.42% and 13.71%, respectively. In terms of work experience, over 64% of respondents have less than 10 years in their roles, indicating a relatively young workforce. Nearly 80% employees hold at least a bachelor’s degree, and 11.24% possess a Ph. D. Most firms are small to medium-sized, with 89.43% employing fewer than 80 people. Industry representation is nearly balanced, though manufacturing (52.81%) slightly exceeds services (47.19%).

In addition, all study variables were positively and significantly correlated at the 0.01 level (Table 2). Pro-environmental behavior was moderately correlated with environmental passion (0.415), green rewards (0.475), and AI literacy (0.410), indicating preliminary support for the hypothesized relationships. These correlations are descriptive, and the main hypotheses were tested using PLS-SEM.

**Table 2:** Correlation test results.

Variable	AI	EP	GR	PEB
AI	1			
EP	0.440***	1		
GR	0.438***	0.520***	1	
PEB	0.410***	0.415***	0.475***	1

Sources: summary by the author. “***”means the result is significant at the 1% level.

### 4.2 Results of PLS-SEM

This paper evaluates the statistical quality of pro-environmental behaviors, environmental passion, green rewards, and AI literacy by examining their factor loadings, reliability, convergent validity, and collinearity. Table 3 describes all results of outer loading, average variance extracted (AVE), Cronbach’s alpha (CA), composite reliability (CR), and collinearity statistics (VIF).

**Table 3:** Factor loadings results.

Construct	Items	Loading	AVE	CA	CR	VIF
Pro-environmental behavior (PEB)	PEB1	0.836	0.686	0.909	0.929	2.314
	PEB2	0.849				2.471
	PEB3	0.819				2.195
	PEB4	0.838				2.416
	PEB5	0.801				2.037
	PEB6	0.825				2.250
Environmental passion (EP)	EP1	0.805	0.667	0.929	0.941	2.249
	EP2	0.817				2.433
	EP3	0.823				2.435
	EP4	0.826				2.476
	EP5	0.815				2.321
	EP6	0.817				2.370
	EP7	0.817				2.365
	EP8	0.815				2.378
Green rewards (GR)	GR1	0.842	0.694	0.892	0.919	2.215
	GR2	0.834				2.131
	GR3	0.829				2.229
	GR4	0.829				2.163
	GR5	0.831				2.071
AI literacy (AI)	AI1	0.772	0.630	0.943	0.949	2.108
	AI2	0.794				2.304
	AI3	0.807				2.397
	AI4	0.815				2.473
	AI5	0.827				2.609
	AI6	0.783				2.241
	AI7	0.792				2.282
	AI8	0.803				2.383
	AI9	0.771				2.120
	AI10	0.782				2.220
	AI11	0.780				2.167

Sources: summary by the author.

All item loadings exceed the threshold of 0.7 (Hair et al., 2022b), indicating strong relationships between each item and its corresponding construct. For instance, pro-environmental behavior items range from 0.801 to 0.849. CA values are all above 0.89, exceeding the recommended threshold of 0.7 (David et al., 2024). This value indicated excellent internal consistency. CR values range from 0.919 to 0.949, all exceeding the critical value of 0.7 and further confirming reliability (Misa et al., 2024). In addition, AVE values are all above 0.63, exceeding the threshold of 0.5 and confirming that each construct adequately explains the variance of its items (David et al., 2024).

Furthermore, the maximum of VIF is 2.609 for AI5. All VIF values are below 3.0, which satisfies the conservative threshold of 5.0 (Darwis et al., 2025). This indicates no significant multicollinearity among items within constructs. In addition, Harman’s single-factor test was conducted to assess potential common method bias. Harman test is an unrotated factor analysis and shows that no single factor accounted for the majority of variance, indicating that CMB was unlikely to be a major concern (Jantapoon, 2025). The results of this study indicate that the first factor explains 39.79% of the total variance, which is below the widely accepted threshold of 40%. Both results of Harman’s single-factor test combined with VIF values suggest that common method bias is unlikely to be a concern in this study.

Table 4 shows the results of the Heterotrait–Monotrait Ratio (HTMT). All HTMT ratios are aligning with the citric value of 0.9, which typically corroborates discriminant validity (Haque et al., 2024).

**Table 4:** HTMT results.

Variable	HTMT ratio	Interpretation
EP and AI	0.482	Acceptable
EP and GR	0.563	Acceptable
EP and PEB	0.483	Acceptable
GR and AI	0.490	Acceptable
GR and PEB	0.522	Acceptable
AI and PEB	0.446	Acceptable

Sources: summary by the author.

Table 5 lists the Fornell–Larcker criterion value. The Fornell–Larcker criterion evaluates discriminant validity in SEM, confirming that constructs are distinct from one another (Haque et al., 2024). All results align with the criterion that the square root of AVE or each item exceeds its correlations with other items. Diagonal values in Table 5 represent the square root of AVE for each item.

**Table 5:** Fornell–Larcker criterion results.

Variable	AI	EP	GR	PEB
AI	0.793			
EP	0.453	0.817		
GR	0.452	0.514	0.833	
PEB	0.414	0.444	0.473	0.828

Sources: summary by the author.

Combining the results of Tables 3–5, this paper demonstrated great reliability and convergent validity. The reliability and convergent validity for Chinese contexts were indicated by the results of AVE, CA, CR, VIF, HTMT, and Fornell–Larcker.

Then, this study utilized PLS-SEM to analyze the hypothesized associations. The results demonstrate support for all proposed hypotheses across the overall sample and within the manufacturing and service sectors (Table 6). Table 6 presents descriptive statistics for respondents across sectors. “Whole sectors” includes all respondents from both manufacturing and service sectors. “Manufacturing sectors” and “Service sectors” refer to employees from these broad categories. The results indicated that the *p*-value for each hypothesis on whole sectors was below 0.05, supporting the hypothesis.

**Table 6:** Outcomes of hypotheses (direct and indirect effects).

Hypothesis	Path	Coefficients	Result	Coefficients	Result	Coefficients	Result
		Whole sectors		Manufacturing sector		Service sector	
H1	GR → PEB	0.293 (0.000)	Support	0.279 (0.000)	Support	0.321 (0.000)	Support
H2a	GR → EP	0.393 (0.000)	Support	0.340 (0.000)	Support	0.450 (0.000)	Support
H2b	EP → PEB	0.183 (0.001)	Support	0.213 (0.003)	Support	0.153 (0.067)	Support
Outcomes of hypotheses (indirect effects)							
H2: GR → EP → PEB		0.072 (0.002)	Support	0.072 (0.014)	Support	0.110 (0.082)	Support
H3: AI* GR → PEB		0.201 (0.000)	Support	0.112 (0.063)	Support	0.229 (0.001)	Support
H4: AI* GR → EP → PEB		0.031 (0.016)	Support	0.185 (0.004)	Support	0.157 (0.035)	Support

Sources: summary by the author. “Whole sectors” refers to all respondents from both manufacturing and service sectors. “Manufacturing sectors” and “Service sectors” indicate respondents from enterprises in these broad categories, without further subdivision into specific industries.

For direct effects, green rewards showed a positive effect on pro-environmental behaviors in the whole sample (0.293, *p* < 0.001), the manufacturing sector (0.279, *p* < 0.001), and the service sector (0.321, *p* < 0.001). This indicated a consistent and positive relationship between green rewards and pro-environmental behaviors across sectors, supporting H1. In addition, green rewards may significantly enhance environmental passion in all groups, with the strongest effect in the service sector (0.450, *p* < 0.001). H2a was supported across all groups. Then, environmental passion has a positive relationship with pro-environmental behaviors, supporting H2b. environmental passion positively predicted pro-environmental behaviors in the whole sample (*β* = 0.183, *p* = 0.001) and the manufacturing sector (*β* = 0.213, *p* = 0.003). In the service sector, the effect was positive but marginally significant (*β* = 0.153, *p* = 0.067).

For indirect effects, this paper examined the moderating effect of AI literacy and the mediating effect of environmental passion. First, environmental passion mediates the relationship between green rewards and pro-environmental behaviors in the whole sample (*β* = 0.072, *p* = 0.002) and manufacturing (*β* = 0.072, *p* = 0.014). In the service sector, the mediation was marginally significant (*β* = 0.110, *p* = 0.082). Thus, H2 is supported overall and partially supported in the subgroup analysis. For moderation effects, H3 is supported. AI literacy strengthens the relationship between green rewards and pro-environmental behavior, particularly in the service sector (*β* = 0.229, *p* = 0.001) compared to manufacturing (*β* = 0.112, *p* = 0.063). Furthermore, AI literacy also enhances the indirect pathway through environmental passion, with the largest moderating effect in manufacturing (*β* = 0.185, *p* = 0.004). This finding supported H4.

### 4.3 ANN analysis results

The RMSE results in Table 7 assess the ANN’s predictive performance for pro-environmental behaviors across the whole sample, the manufacturing sector, and the service sector. All mean RMSE of training tests across the whole sample, the manufacturing sector, and the service sector remained low, indicating that the model fits the training data well. Testing errors are slightly higher than training errors in the whole sample and the manufacturing sector, reflecting moderate prediction accuracy but no major overfitting (Guo et al., 2025). This divergence between training and testing errors suggests that the determinants of pro-environmental behavior exhibit contextual complexity, particularly when moving from model learning to out-of-sample prediction. This sector shows a higher testing error than training error, indicating variability or complexity in predicting pro-environmental behavior in the service context. This finding implies that pro-environmental behavior in service settings may be driven by more heterogeneous and non-linear mechanisms than those captured through linear modeling alone. The standard deviations (SD) for both training and testing errors are small across all models, indicating stable performance and consistent convergence.

**Table 7:** RMSE values for testing PEB.

Input neurons	GR, EP, AI*GR					
Output nodes	PEB (whole)		PEB (manufacturing)		PEB (service)	
Neural network	Training	Testing	Training	Testing	Training	Testing
1	0.043	0.107	0.058	0.151	0.064	0.204
2	0.042	0.109	0.058	0.196	0.061	0.192
3	0.040	0.140	0.056	0.163	0.062	0.177
4	0.042	0.107	0.056	0.143	0.063	0.172
5	0.042	0.111	0.056	0.175	0.063	0.155
6	0.042	0.131	0.062	0.202	0.061	0.170
7	0.041	0.122	0.058	0.165	0.062	0.145
8	0.041	0.131	0.058	0.164	0.061	0.192
9	0.043	0.108	0.060	0.169	0.063	0.162
10	0.042	0.119	0.057	0.141	0.062	0.168
Mean	0.042	0.118	0.058	0.167	0.062	0.174
SD	0.001	0.012	0.002	0.020	0.001	0.018

Sources: summary by the authors. The abbreviation of ‘standard deviation’ is SD. “Whole sectors” refers to all respondents from both manufacturing and service sectors. “Manufacturing sectors” and “Service sectors” indicate respondents from enterprises in these broad categories, without further subdivision into specific industries.

The RMSE results in Table 8 evaluate the ANN’s performance in predicting environmental passion. All low training errors but slightly higher testing errors indicated moderate prediction accuracy. Small SD across all models reflects stable and reliable performance. These results suggest that environmental passion formation process may involve non-linear dynamics that vary across sectors. The non-linear dynamics reinforce the value of ANN in uncovering latent behavioral complexity beyond linear SEM-based explanations.

**Table 8:** RMSE values for testing environmental passion.

Input neurons	GR, AI*GR					
Output nodes	EP (whole)		EP (manufacturing)		EP (service)	
Neural network	Training	Testing	Training	Testing	Training	Testing
1	0.041	0.119	0.058	0.160	0.059	0.140
2	0.041	0.120	0.059	0.142	0.060	0.143
3	0.041	0.119	0.057	0.172	0.060	0.150
4	0.042	0.105	0.058	0.149	0.059	0.141
5	0.042	0.122	0.057	0.184	0.058	0.196
6	0.042	0.113	0.060	0.141	0.059	0.146
7	0.042	0.099	0.057	0.160	0.058	0.172
8	0.040	0.123	0.056	0.204	0.058	0.188
9	0.042	0.114	0.060	0.129	0.060	0.161
10	0.041	0.122	0.058	0.144	0.061	0.144
Mean	0.041	0.116	0.058	0.159	0.059	0.158
SD	0.001	0.008	0.001	0.023	0.001	0.020

Sources: summary by the authors. The abbreviation of ‘standard deviation’ is SD. “Whole sectors” refers to all respondents from both manufacturing and service sectors. “Manufacturing sectors” and “Service sectors” indicate respondents from enterprises in these broad categories, without further subdivision into specific industries.

### 4.4 Comparison between PLS-SEM and ANN results

Table 9 compares the importance of factors affecting pro-environmental behaviors and environmental passion using PLS-SEM and ANN.

**Table 9:** Comparison of factor importance between PLS-SEM and ANN results.

Comparation	Path coefficient (PLS-SEM)			Normalized importance (ANN)			Ranking (PLS-SEM)			Ranking (ANN)		
	W	M	S	W	M	S	W	M	S	W	M	S
Test 1: Output nodes-PEB												
GR → PEB	0.293	0.279	0.321	100%	100%	100%	1	1	1	1	1	1
EP → PEB	0.183	0.153	0.153	89%	95%	64%	3	3	2	2	2	2
AI*GR → PEB	0.201	0.229	0.229	62%	55%	54%	2	2	3	3	3	3
Test 2: Output nodes-EP												
GR → EP	0.393	0.34	0.45	100%	100%	100%	1	1	1	1	1	1
AI*GR → EP	0.031	0.185	0.157	65%	90%	35%	2	2	2	2	2	2

Sources: summary by the authors. The abbreviations of the whole sector, the manufacturing sector, and the service sector are W, M, and S. AI*GR refers to the interactive term of AI literacy aligning with green rewards. “W” refers to all respondents from both manufacturing and service sectors. “M” and “S” indicate respondents from enterprises in these broad categories, without further subdivision into specific industries.

The comparative results demonstrate that green rewards are the most critical factor influencing both pro-environmental behaviors and environmental passion across all sectors, consistently ranking first in both methods. Based on the significance and relationship direction identified through PLS-SEM, the ANN analysis further reveals its dominant relative importance within the overall theoretical structure. For pro-environmental behaviors, green rewards are followed by environmental passion and the interaction term of AI and green rewards. In contrast to PLS-SEM path coefficients, ANN revealed the relative predictive importance of each variable, with the standardized relevance of the interaction term of AI and green rewards varying between 54% and 62%. This variation highlights the non-linear and contingent role of AI-enabled green rewards mechanisms, which may not be fully captured by linear interaction effects alone. Similarly, green rewards remain dominant for environmental passion, followed by the interaction term of AI and green rewards. The differences among sectors in the rankings are minimal yet significant in magnitude. This indicates that the overall theoretical relationships remain stable, but the strength and significance of specific drivers vary across sectoral contexts.

## 5 Discussion

The results from both PLS-SEM and ANN analyzes provide robust support for the hypothesized relationships. The ANN analysis provides additional theoretical insights beyond the PLS-SEM results. PLS-SEM confirms the existence and direction of hypothesized relationships. The ANN model reveals the relative importance and potential non-linear influence of key predictors. These findings suggest that certain constructs play a more central role within the theoretical mechanism than implied by linear modeling alone. By integrating explanatory and predictive perspectives, this combined approach advances theory by moving beyond hypothesis testing toward a deeper understanding of underlying behavioral complexity.

First, green rewards exhibit a significant positive effect on pro-environmental behaviors, supporting Hypothesis 1. This suggests that organizational green incentives could effectively motivate employee environmental actions because they enhance perceived value of employees and reinforce recognition of pro-environmental behavior. Employees may respond positively to tangible rewards as these align personal interests with organizational environmental goals. This finding aligns with the finding of Wang and Guo (2025) but contrasts with the finding of Maris et al. (2025). Such discrepancies may be attributed to contextual differences, including organizational culture, reward design, and implementation mechanisms, which can moderate the effectiveness of incentive-based strategies.

Second, H2a is supported by the positive relationship between green rewards and environmental passion. This indicates that green rewards directly improve overall environmental passion by fostering employee engagement and commitment to sustainability practices. This confirmed the finding of Yang et al. (2024) but refuted the finding of Nyanghura et al. (2024). Similarly, H2b is supported by the positive relationship between environmental passion and pro-environmental behaviors. This implies that higher environmental passion creates a reinforcing feedback loop, where employees are more likely to continue pro-environmental behaviors because they perceive tangible improvements and organizational acknowledgment. This finding is contrast to the findings of Wang and Li (2024) and Wang D. et al. (2023). In addition, the mediated role of environmental passion is validated, indicating that green rewards indirectly influence pro-environmental behaviors through internal psychological states. This finding supported the H2 and the research of Maleknia and Enescu (2025) related to Stimulus-Organism-Response theory. Green rewards act as external stimuli that enhance organizational processes (environmental passion), which in turn shape employee responses (pro-environmental behaviors).

Third, AI literacy strengthens the relationship between green rewards and pro-environmental behaviors, supporting H3. This provides empirical support for the Social Cognitive theory. The moderating effect is particularly significant in the service sector. This suggests that employees with higher AI literacy can more efficiently apply digital tools to translate incentives into actionable pro-environmental behaviors. Essentially, AI literacy enhances employees’ self-efficacy and capacity to act on organizational incentives, which explains the stronger effect observed. This finding confirmed the findings of Muawanah et al. (2024) and was against the views of Desroches et al. (2025).

Fourth, AI literacy may enhance the indirect pathway from green rewards through environmental passion to pro-environmental behavior, validating H4. This suggests that employees with better AI skills are more capable of converting organizational incentives into improved environmental performance, which subsequently fosters pro-environmental behaviors. The strongest moderating effect in the manufacturing sector indicates that structured workflows and process-oriented environments allow AI-driven engagement tools to be most effective, highlighting the role of organizational context in moderating digital capability impacts. This finding aligns with Arif et al. (2025) but is against the finding of Ruiu et al. (2025).

Furthermore, both models identify green rewards as the most critical driver of pro-environmental behaviors and environmental passion. This emphasizes that tangible incentives remain a key mechanism through which organizations can influence behaviors and passion. The normalized importance results derived from ANN confirmed the robustness of these relationships, and demonstrate how ANN contributes additional insight by differentiating the salience of predictors rather than merely confirming their effects. This consistent findings between explanatory and predictive analyses fill the gap identified by the research of San Román-Niaves et al. (2025), which have focused excessively on direct effects at the expense of in-depth exploration of causal pathways. Overlooking the potential mechanisms and possible contextual factors that may enhance or diminish their efficacy.

### 5.1 Conclusion

This study strengthens the theoretical contribution by clarifying how green rewards influence pro-environmental behaviors. The findings show that green rewards function both as a direct driver of behavior and through the mediating role of environmental passion. This dual influence provides robust empirical validation for the Stimulus-Organism-Response framework in manufacturing and service contexts. In addition, the finding that AI literacy significantly moderates these relationships extends Social Cognitive theory. It reveals that employees with higher digital skills exhibit greater responsiveness to green incentives and more capable of translating them into sustainable actions. These insights help clarify inconsistencies in prior literature. Differences in employees’ digital capability may have shaped whether incentives were effectively translated into behavior. By integrating these insights, this study offers a comprehensive explanation that highlights the joint impact of incentives, emotional mechanisms, and digital skills on pro-environmental behavior.

Findings of this paper offer clear empirical guidance for organizations aiming to foster sustainability. Firstly, integrating green rewards into workplace policies can directly encourage pro-environmental behaviors and cultivate a sense of environmental responsibility. Secondly, enhancing employees’ AI literacy can significantly boost the efficacy of reward systems by improving their interaction with digital tools and sustainability platforms. Organizations should also consider embedding sustainability into corporate culture, reinforcing pro-environmental values through leadership practices and internal communication. In addition, managers could adopt diverse incentive mechanisms across different sectors to enhance different employee motivations, including monetary, non-monetary, and team-based rewards. Lastly, policymakers and managers should focus on sector-specific incentives frameworks to enhance pro-environmental behaviors. Service industries can leverage digital engagement tools, while manufacturing firms can embed AI-driven incentives into operational workflows to maximize behavioral impact. To ensure long-term effects, organizations should implement monitoring and feedback systems that track environmental behaviors over time. Policymakers can support this by establishing industry standards or guidelines. These practical insights provide clear guidance for managers and policymakers on how to tailor incentives, skill-building initiatives, and organizational strategies to promote consistent and sustainable pro-environmental behavior.

This study provides a methodological contribution through the combined use of PLS-SEM and ANN. PLS-SEM enables testing of hypothesized relationships and examination of mediating and moderating mechanisms. ANN captures potential nonlinear patterns and enhances predictive accuracy. By integrating these two approaches, the study improves the measurement and understanding of complex constructs such as pro-environmental behaviors and strengthens the robustness of empirical findings. This dual-method design offers a framework for future research seeking to balance explanatory power with predictive performance.

### 5.2 Future directions

Future studies may broaden these findings in various directions. First, this study identified the moderating effect of AI literacy but did not investigate the particular factors driving this influence. Future studies should concentrate on the specific components of AI literacy that influence the relationship between green rewards and pro-environmental behaviors, such as digital skills, data interpretation, or technology adoption.

Second, this study examined variables’ correlations via cross-sectional surveys. Future studies may include temporal aspects to conduct longitudinal analyses, which could discover long-term interplay between green incentives and AI literacy in modifying environmental behaviors.

Third, although passion and rewards were shown to influence pro-environmental behaviors, future research could investigate additional emotional, cognitive, and incentive mechanisms. For instance, future study could analyze how intrinsic and extrinsic motivators interact over time, or whether certain reward structures sustain long-term environmental engagement.

Fourth, this study concentrated on China’s manufacturing and service industries. Subsequent analyses may expand the scope to encompass additional cultural and industrial contexts to improve the generalizability of the results and assess context-specific mechanisms. Expanding analyses could uncover context-specific drivers of pro-environmental behaviors.

Fifth, researcher reflexivity should be incorporated by critically reflecting on how study design, measurement choices, and theoretical assumptions influence findings, enabling more nuanced interpretations and responsible application of results. Future studies can build on the combined use of PLS-SEM and ANN by paying careful attention to model specification, adequate sample size, and overfitting prevention. This dual-method approach allows researchers to examine both structural relationships and nonlinear predictive patterns, providing a more comprehensive understanding of complex behavioral mechanisms. Researchers can also include additional behavioral or contextual variables to further explore pro-environmental behavior.

Finally, future studies should consider ethical aspects, platform and data usage, and organizational factors that may influence the applicability of results. By applying this approach, future studies can address limitations of prior research that relied on a single analytical technique and improve the measurement and interpretation of key constructs, thereby advancing understanding of the drivers of pro-environmental behavior.

## Data Availability

The original contributions presented in the study are included in the article/supplementary material, further inquiries can be directed to the corresponding author.
