# Supplementary material for: Environmental passion and AI literacy shape the impact of green rewards on pro-environmental behaviors
Source: Front Psychol. 2026 Feb 25;17:1699919. doi: 10.3389/fpsyg.2026.1699919 (PMC12975559; doi:10.3389/fpsyg.2026.1699919)
Supplement: Supplementary file 1 [file Data_Sheet_1.docx]

Supplementary Materials

1. Questionnaire items

|  | Items |
| --- | --- |
| PEB1 | I prevent unnecessary printing to save papers |
| PEB2 | I use personal cups instead of disposable cups |
| PEB3 | I choose stairs instead of elevators when going from floor to floor within a building |
| PEB4 | I reuse papers to take notes in the office |
| PEB5 | I recycle reusable things in the workplace |
| PEB6 | I sort recyclable materials into appropriate bins when others do not. |
| EP1 | I am passionate about the environment |
| EP2 | I enjoy practicing environmentally friendly behaviors |
| EP3 | I enjoy engaging in environmentally friendly behaviors |
| EP4 | I take pride in helping the environment |
| EP5 | I get pleasure from taking care of the environment |
| EP6 | I passionately encourage others to be more environmentally responsible |
| EP7 | I have voluntarily donated time or money to help the environment in some way |
| EP8 | I feel strongly about my environmental values |
| GR1 | The company provides non-financial rewards for good employee environmental performance |
| GR2 | Companies are able to appreciate the environmental performance of employees |
| GR3 | Companies can reward financially for good employee environmental performance |
| GR4 | Give praise to employees who have done a good job. |
| GR5 | Institute link suggestion schemes into reward system by introducing rewards for innovative environmental initiative performance. |
| AL1 | I can distinguish between smart devices and non-smart devices. |
| AL2 | I do not know how AI technology can help me. ^R^ |
| AL3 | I can identify the AI technology employed in the applications and products I use. |
| AL4 | I can skillfully use AI applications or products to help me with my daily work. |
| AL5 | It is usually hard for me to learn to use a new AI application or product. ^R^ |
| AL6 | I can use AI applications or products to improve my work efficiency. |
| AL7 | I can evaluate the capabilities and limitations of an AI application or product after using it for a while. |
| AL8 | I can choose a proper solution from various solutions provided by a smart agent. |
| AL9 | I can choose the most appropriate AI application or product from a variety for a particular task. |
| AL10 | I always comply with ethical principles when using AI applications or products. |
| AL11 | I am never alert to privacy and information security issues when using AI applications or products. ^R^ |

2. Questionnaire sheets

The data that support the findings of this study are openly available in Zenodo at <https://doi.org/10.5281/zenodo.17751655.> The dataset has been anonymized to protect participant confidentiality.
